# Supplementary material for: Quantitative proteomics reveals tissue-specific, infection-induced and species-specific neutrophil protein signatures
Source: Sci Rep. 2024 Mar 12;14:5966. doi: 10.1038/s41598-024-56163-6 (PMC10933280; doi:10.1038/s41598-024-56163-6)

Supplementary Figure 1 – Gating strategy for fluorescent activated cell sorting pure neutrophil populations from mouse bone marrow, blood and peritoneal cavity and from human blood. Mouse neutrophil populations were stained with Ly-6G FITC, CD11b APC and DAPI and a pure population of neutrophils (Ly6G and CD11b high) were isolated by cell sorting. Human blood neutrophils were sorted on the basis of size, granularity, and autofluorescence based on forward/side scatter and autofluorescence.

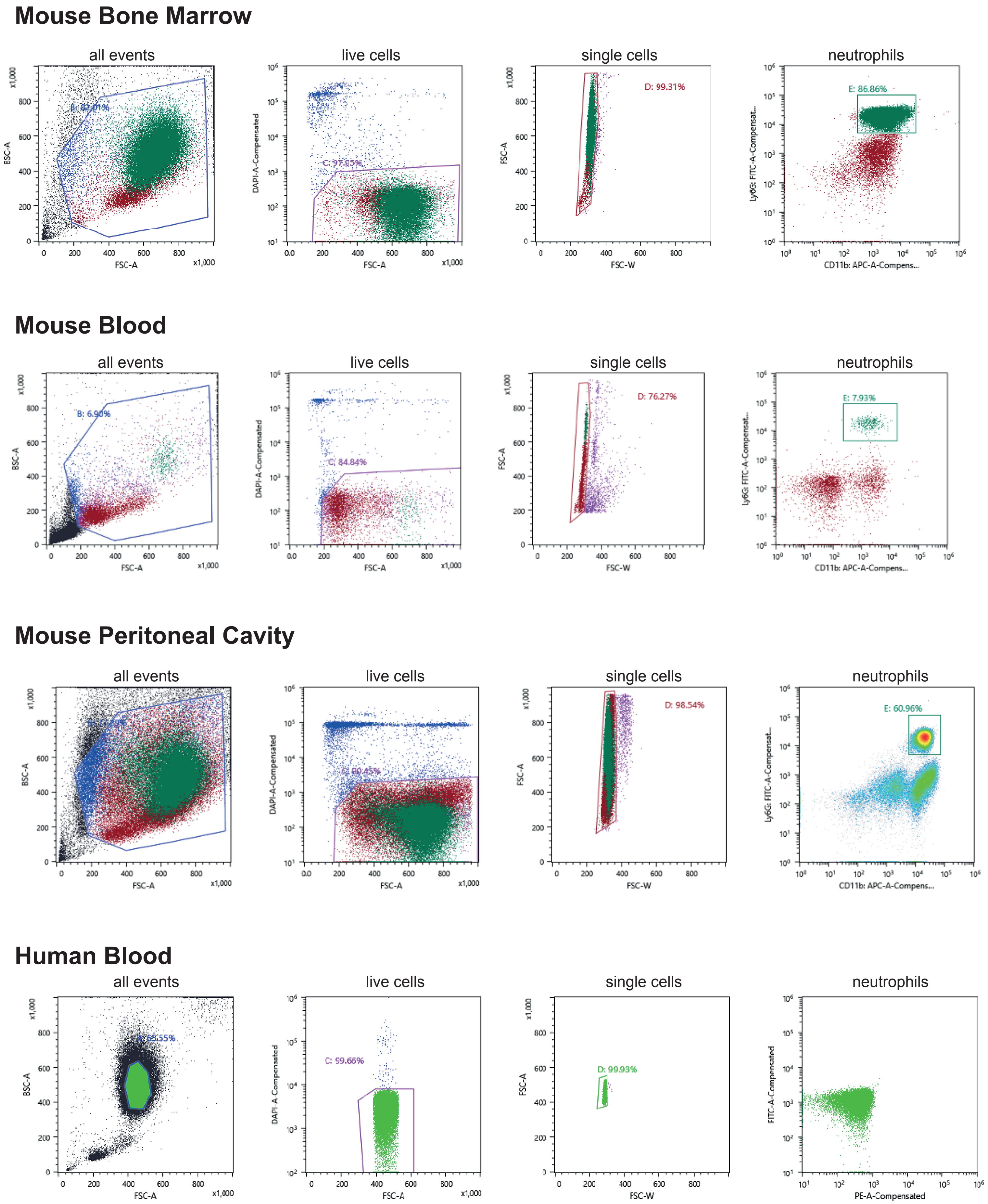

Supplementary Figure 2 – Overview of mouse bone marrow, blood and peritoneal cavity neutrophil proteomes. Each protein is represented by a circle, with circle size indicating relative abundance. Granule proteins are subdivided into subsets while non-granule proteins are coloured grey.

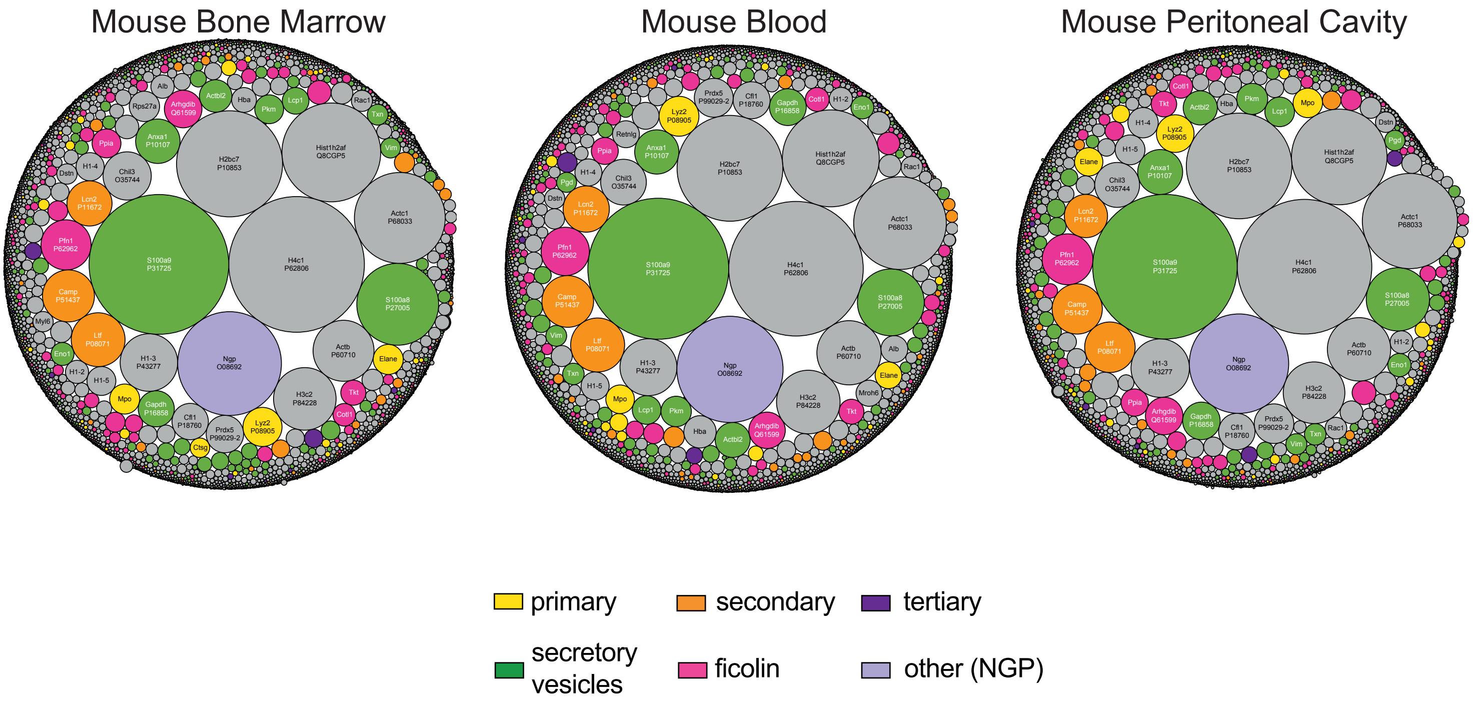

Supplementary Figure 3 – The expression profile of transcription factors in mouse neutrophil populations. Volcano plots show the expression profile of proteins annotated as having DNA binding or transcription factor activity (Gene Ontology term 0003700). For each population 4 biological replicates were generated. BM: bone marrow, BL: blood, PT: peritoneal cavity. The horizontal dashed line on volcano plots indicates q = 0.05.

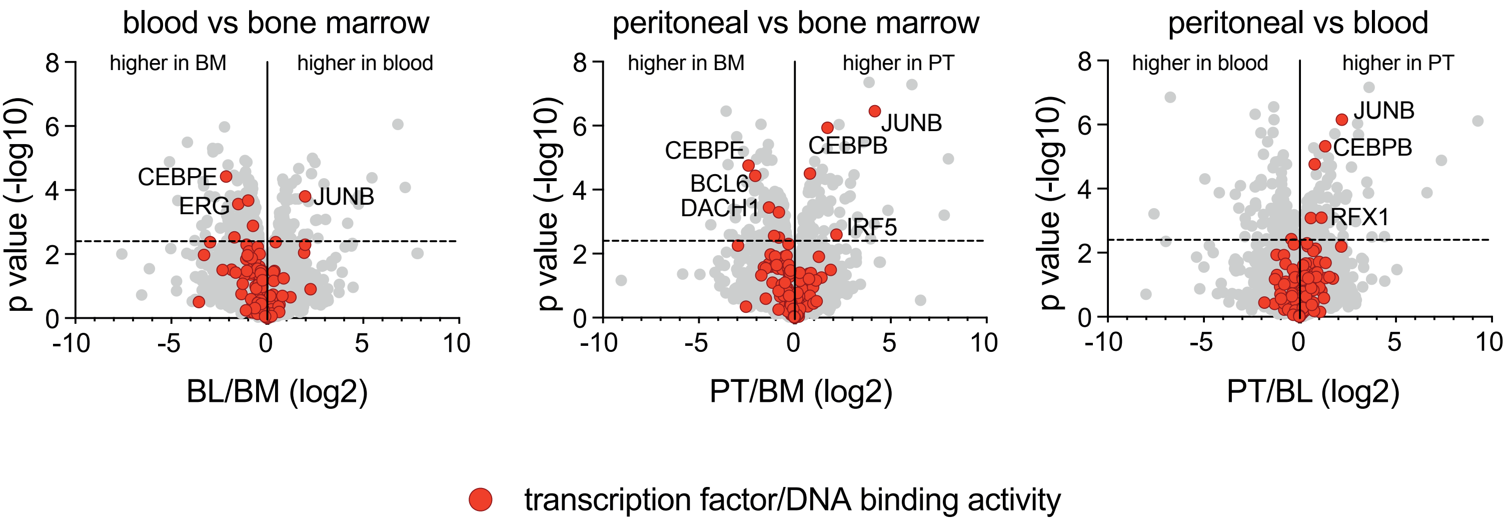

Supplementary Figure 4 – Gating strategy for fluorescent activated cell sorting pure neutrophil populations from *Candida albicans* infection experiment. Neutrophils from infected or control mice (PBS treated) were sorted using Ly-6G FITC, CD11b PE-Cy7 and DAPI.

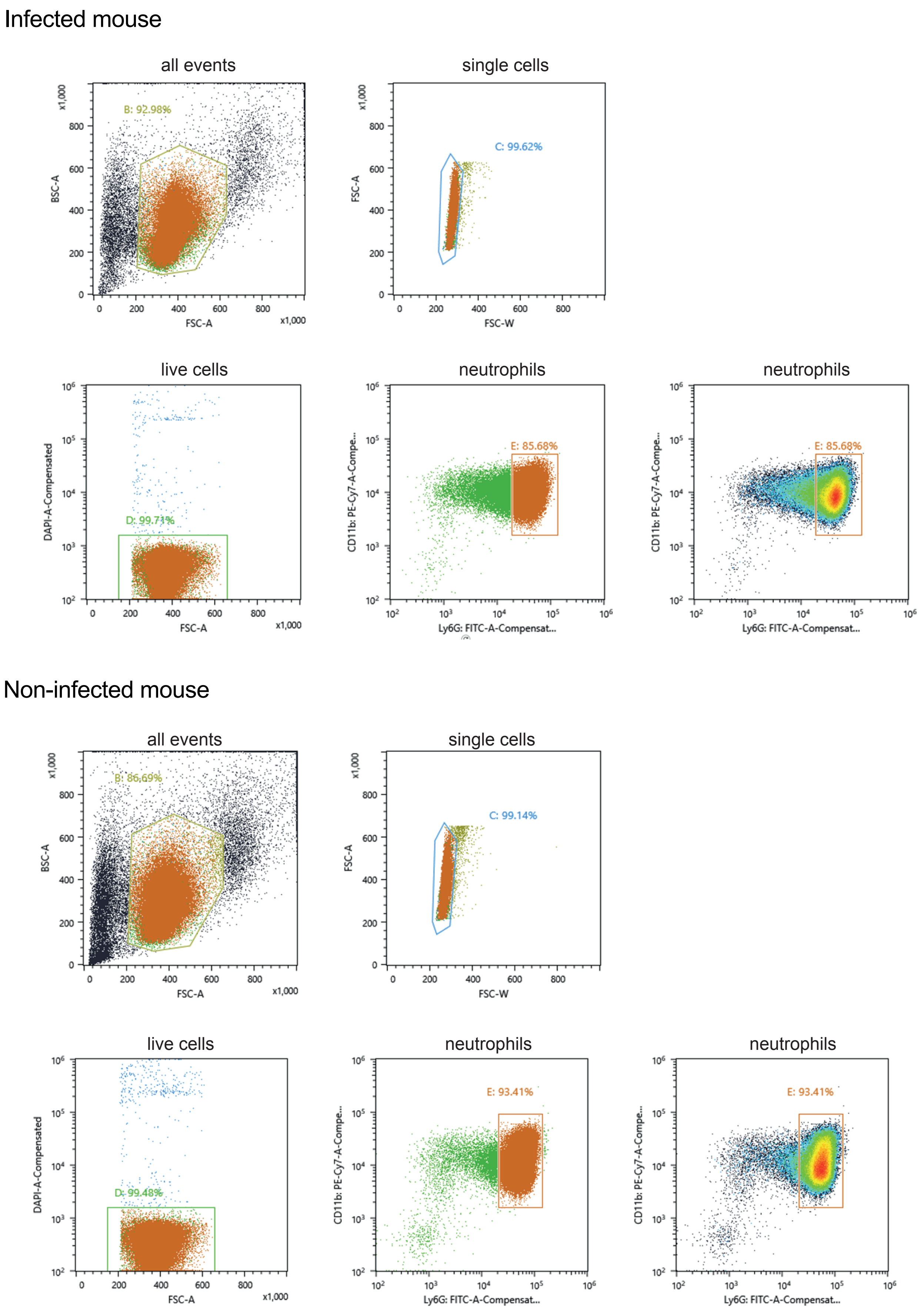

Supplementary Figure 5 – The impact of systemic infection on bone marrow neutrophil DNA replication machinery. Schematic showing the DNA replication fork complex and the average protein copy numbers per cell for infected and non-infected neutrophils. Copy numbers are rounded to the nearest thousand except DNA2 which is rounded to the nearest hundred. For each population 3 biological replicates were generated.

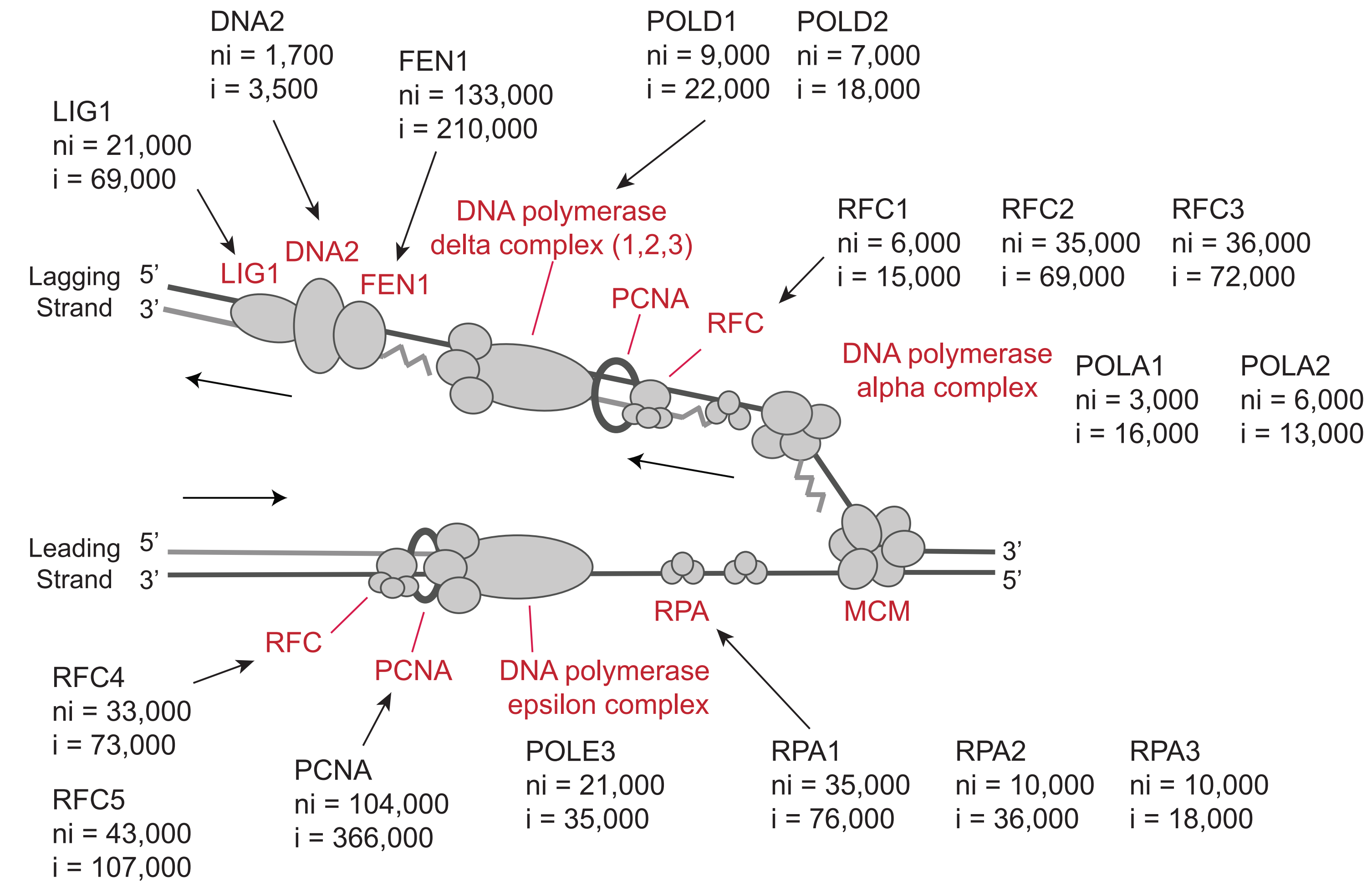

Schematic of replication fork complex adapted from Damasio et al., 2021

ni = non-infected
i = infected

Supplementary Figure 6 – The impact of systemic infection on CEBP transcription factors. Dot plots show the mean +/- standard deviation. Statistical significance was determined using Limma with eBayes. * indicates p<0.05. CCAAT Enhancer Binding Protein Beta (CEBPB), CCAAT Enhancer Binding Protein Delta (CEBPD) and CCAAT Enhancer Binding Protein Epsilon (CEBPE). CEBPB was detected in only 2 biological replicates from infected mice and 2 biological replicates from non-infected mice.

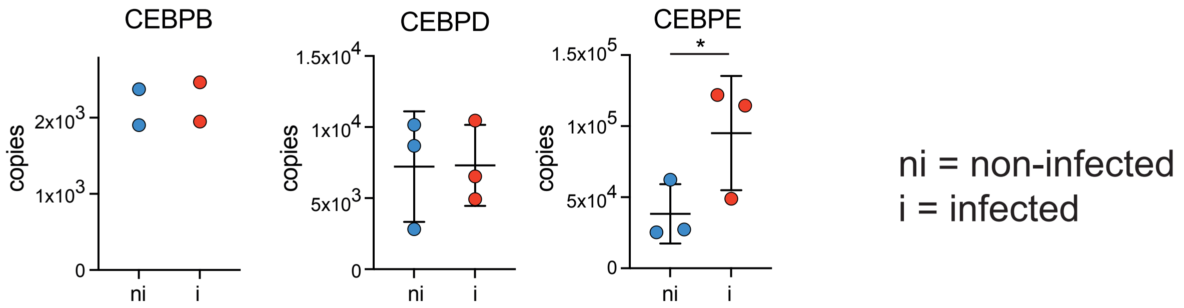

Supplement: Supplementary file 1 — Supplementary Figures. [file 41598_2024_56163_MOESM1_ESM.pdf]
