# Supplementary material for: Quantitative proteomics reveals tissue-specific, infection-induced and species-specific neutrophil protein signatures
Source: Sci Rep. 2024 Mar 12;14:5966. doi: 10.1038/s41598-024-56163-6 (PMC10933280; doi:10.1038/s41598-024-56163-6)
Supplement: Supplementary file 3 — Supplementary Table 1. [file 41598_2024_56163_MOESM3_ESM.pdf]

Supplementary Table 1 – The expression of 6 platelet-associated proteins in neutrophils isolated from mouse blood. Copy numbers = average copy number per cell, relative abundance = (copy number/sum of all protein molecules within the cell)*100. The proteins listed are platelet-associated and may also be expressed by other cells.

Gene Name	Protein Name	Copy Numbers	Relative Abundance
CD36	Platelet glycoprotein 4	not detected	-
SELP	P-selectin	not detected	-
CD31/PECAM1	Platelet And Endothelial Cell Adhesion Molecule 1	not detected	-
CD41/ITGA2B	Integrin Subunit Alpha 2b	6,700	<0.001%
GP1BA	Glycoprotein Ib Platelet Subunit Alpha	1,300	<0.0001%
GP1BB	Glycoprotein Ib Platelet Subunit Beta	5,800	<0.001%
